# Enhanced Recovery After Surgery (ERAS): New Concepts in the Perioperative Management of Gynecologic Surgery

**DOI:** 10.1055/s-0038-1668581

**Published:** 2018-08

**Authors:** Agnaldo Lopes da Silva Filho, Aline Evangelista Santiago, Sophie Françoise Mauricette Derchain, Jesus Paula Carvalho

**Affiliations:** 1 Universidade Estadual Paulista “Júlio de Mesquita Filho,” Botucatu, SP, Brazil; 2 Universidade Federal de Minas Gerais, Belo Horizonte, MG, Brazil; 3 Universidade Estadual de Campinas, Campinas, SP, Brazil; 4 Universidade de São Paulo, São Paulo, SP, Brazil

## What is ERAS?

The Enhanced Recovery After Surgery (ERAS) program is a paradigm shift from traditional perioperative management initiated by Kehlet in 1997^1^ as a multidisciplinary approach to the care of the surgical patient.^1^ ^2^ ^3^ The program is based on perioperative medical optimization, including preoperative counseling, pain relief, carbohydrate loading, thromboembolism prophylaxis, standard anesthetic protocol and intraoperative fluid, recovery of normal gastrointestinal function, and early mobilization (Table 1). The primary goal of the protocol is to minimize the response to the stress of the operation by maintaining homeostasis, avoiding catabolism with consequent loss of protein and muscle strength, and cellular dysfunction.^3^

**Table 1:** Enhanced Recovery After Surgery (ERAS) program principles

Enhanced Recovery After Surgery (ERAS) program		
What does it promote?	Why should it be implemented?	What is necessary for the implementation?
– Minimization of the stress response to the operation by controlling the perioperative physiology – Operative medical optimization: pre-operative counseling, pain relief, carbohydrate loading, thromboembolism prophylaxis, standard anesthetic protocol and intraoperative fluid, recovery of normal gastrointestinal function, and early mobilization	– Shorter length of hospital stay – No increase in readmissions and/or reoperations and/or complications rates – Faster and safer patient recovery – Improved quality of life and patient satisfaction – Reduction in overall healthcare costs	– Program coordinator (doctor/nurse) – Involvement of all units dealing with the surgical patient – Multidisciplinary team working together around the patient – Multimodal approach to resolving issues that delay recovery and cause complications – Scientific, evidence-based approach to care protocols – Change in management through interactive and continuous audits – Whenever possible, minimally invasive surgery

**Source:** Adapted from Kehlet (1997)^1^ and Carey and Molder (2018).^2^

The main objectives of the ERAS program are to accelerate functional recovery, improve postoperative outcomes, shorten the length of stay (LOS) in the hospital, reduce the overall health care costs, and improve the satisfaction of the patients without increasing complications and/or hospital readmission rates.^4^ The ERAS protocols resulted in a 30% to 50% reduction in the LOS and similar reductions in complications, as well as lower costs and readmission rates.^3^ The protocols were developed for colorectal surgery, and variations are being adopted for surgical procedures of various specialties, including Gynecology.^5^ ^6^

The ERAS Society is an international nonprofit professional society that promotes, develops, and implements ERAS programs, publishes updated guidelines for many operations, and was officially registered in 2010 in Sweden (http://erassociety.org). Its mission is to develop perioperative care and to improve recovery through research, education, auditing and implementation of evidence-based practices. Throughout its history, the ERAS Society has developed and published numerous evidence-based protocols and implementation programs worldwide to enhance recovery after surgery. This society conducts structured implementation programs that are currently in use in more than 20 countries. The ERAS Society group published in 2016 the guidelines for pre- and intraoperative care in gynecologic oncology surgery.^7^ ^8^ In 2005, the Department of Surgery of The Faculty of Medical Sciences of Universidade Federal do Mato Grosso, Brazil, adapted the ERAS program to our reality and created the Accelerating the Total Postoperative Recovery (ACERTO, in the Portuguese acronym) project. The application of the ACERTO multimodal protocol determined a significant improvement in morbidity and mortality in general surgery.^9^

## Why should an ERAS pathway be adopted in gynecologic surgeries?

Most of the data on the ERAS program that is available in the literature refers to colorectal surgeries. Variations of the protocol are being adopted for gynecologic procedures despite the limited population and procedure-specific outcome data.^5^ Studies comparing the ERAS program to conventional practices in gynecologic surgery have shown a faster patient recovery, as well as a significant reduction in the LOS without an increase in readmission rates and complications in patients submitted to the practices recommended by the program.^2^ ^3^ ^4^ ^10^ In addition, the incidences of urgent clinic and emergency room visits, readmissions, and reoperations within 90 days of the surgery were similar for patients who were discharged on the day of the surgery compared with those admitted for more than 24 hours.^11^

Introducing the ERAS protocol for abdominal hysterectomy reduced the LOS without increasing complications or readmissions.^10^ For benign vaginal hysterectomies, ERAS has been associated with a reduction in the LOS of 51.6%, and it enables more women to be discharged within 24 hours, with no increase in patient readmissions rates.^12^ Establishing the program for vaginal hysterectomy also resulted in a reduction in costs, coupled with increased patient satisfaction and no rise in morbidity.^13^

In Urogynecology, ERAS implementation has been associated with a greater proportion of same-day discharges and high patient satisfaction, but with slightly increased hospital readmissions within 30 days.^5^ The implementation of ERAS protocols in gynecologic surgeries has been associated with a substantial reduction in the administration of intravenous fluids and morphine, as well as a reduction in the LOS in open procedures associated with improved patient satisfaction and decreased hospital costs.^14^

Regarding minimally invasive surgeries (MISs), increased American Society of Anesthesiologists (ASA) physical status, being African American, and increased length of procedure were significantly associated with readmissions after laparoscopic hysterectomies for benign and malignant conditions performed following an ERAS pathway.^15^ Even in gynecologic oncology MISs in, ERAS is associated with a decreased LOS without increases in morbidity or readmission rates.^11^

The implementation of ERAS protocols for women undergoing major gynecologic surgery has been associated with a substantial decrease in intravenous fluid and morphine administration combined with a reduction in the LOS, improved patient satisfaction, and decreased hospital costs.^14^ Despite the lack of high quality studies evaluating the benefits of enhanced recovery programs in comparison to conventional care for gynecologic cancer patients, this approach is considered a safe perioperative management strategy. The LOS is reduced, without affecting the rates of complications or readmission.^6^ ^16^ ^17^ ^18^

The ERAS principles are applied across all surgical specialties, and ongoing innovation must continue to enable the processes to improve.^3^ A successful ERAS program can lead to a reduction in overall healthcare costs, faster and safer recovery, and improved quality of life and patient satisfaction. In addition, for patients with gynecologic cancer, returning to or getting close to the baseline physiological level enables the accomplishment of the planned adjuvant therapies without delay, resulting in better oncologic outcomes.^4^

## How should an ERAS program be implemented?

The essential aspect in changing the practice and implementing an ERAS pathway is forming a team composed of key individuals from each involved unit.^3^ As illustrated in Table 1, the ERAS program has several principles.^2^ ^3^ The approach to the care of the surgical patient through the various parts of the hospital must be multimodal and multidisciplinary.^3^ The process of implementation of an ERAS program involves a team consisting of surgeons, anesthetists, an ERAS coordinator (often a nurse or a physician assistant), nurses, dieticians, and physiotherapists from units that care for the surgical patient.^3^ No single element by itself will improve the outcomes of surgery. Adherence to the program is crucial, and continuous auditing of the care process enables the team to have a comprehensive view of the patient outcomes.^3^ Minimally invasive surgery is a vital component of an ERAS program, and should be the preferred surgical approach whenever possible.^2^

The program brings together best practices, organization of care and clinical management.^6^ The care protocol is based on published evidence, and it is important to implement additional changes in light of new evidence. An important goal for the ERAS Society is to build a network of hospitals around the world. Successful implementation of an ERAS program requires a multidisciplinary team effort and active participation of the patient in the goal-oriented functional recovery program.^4^ The ERAS program focuses on patients who actively participate in their own recovery process, and ensures they receive adequate postoperative care.

The implementation ERAS in gynecologic surgery involves four essential stages: the preadmission, preoperative, intraoperative, and postoperative stages.^2^ The strategies include preadmission counseling, avoidance of preoperative bowel preparation, use of opioid-sparing multimodal perioperative analgesia (including locoregional analgesia), intraoperative goal-directed fluid therapy, and avoidance of routine use of nasogastric tubes, drains and/or catheters.^4^ Postoperatively, it is important to encourage early feeding, early mobilization, timely removal of tubes and drains, if present, and opioid-sparing analgesia regimens.

The recommendations of the perioperative enhanced recovery pathway for gynecologic surgeries are shown in Table 2.^2^ ^4^ ^7^ ^8^ ^17^ Smoking and alcohol consumption (alcohol abusers) should cease four weeks before surgery. Anemia should be actively identified, investigated, and corrected preoperatively. Mechanical bowel preparation should not be used routinely even when bowel resection is planned. The intraoperative prevention of intraoperative hypothermia with suitable active warming devices should be used routinely. Very restrictive or liberal fluid regimes should be avoided in favor of euvolemia. The intraoperative stage recommendations include the standard anesthetic protocol, avoidance of nasogastric tubes or removal at the end of surgery, and infusion of local anesthetic (bupivacaine) in the wound (deep and superficial injections) prior to closure.^4^ ^7^

**Table 2:** Main recommendations of the perioperative enhanced recovery pathway for gynecologic surgeries

Enhanced Recovery After Surgery (ERAS) program recommendations for gynecologic surgeries		
**Preadmission stage**	Prevention of complications	Appropriate preoperative risk stratification, timely risk modification, and medical optimization have to be performed. Screen and treat anemia
	Counseling	Preoperative counseling of patients and caregivers
**Preoperative stage**	Bowel preparation	Elimination of mechanical bowel preparation and rectal enema for most procedures
	Diet	No solids after midnight; clear liquid diet 2–4 hours before surgery; 100-g carbohydrate-loaded drink the night before surgery; and a 50-g carbohydrate-loaded drink 2–4 hours before surgery
	Premedication	Avoid long- or short-term sedative agents (Tramadol ER, Pregabalin, Celecoxib, Acetaminophen IV)
	IVF therapy	Saline lock until going to the OR
**Intraoperative stage**	Analgesia immediately before going to the OR	Acetaminophen 1,000 mg PO; Gabapentin 600–1,200 mg PO once or Pregabalin 100–300 mg PO once; Celecoxib 200–400 mg PO once
	Nausea and vomiting prophylaxis	Scopolamine transdermal patch 2 hours preoperatively; Dexamethasone 4 mg IV once at induction
	Analgesia	Total intravenous anesthesia; regional anesthesia if appropriate; Acetaminophen 1,000 mg IV once (if not oral); local wound infiltration: preincisional or postincisional bupivacaine hydrochloride or postincision liposomal bupivacaine
	Fluid balance	Goal-directed fluid therapy with a net zero balance at the end of the surgical case; Lactated Ringer's over normal saline for electrolyte balance
**Postoperative stage**	IVF therapy	IVF 40 ml/h; saline lock when tolerating 500 ml oral
	Analgesia	Opioid-sparing analgesia; Acetaminophen or Ibuprofen; Pregabalin, 75 mg every 12 hours (for 48 hours)
	Nausea and vomiting management	Ondansetron 4 mg PO every 6 hours prn nausea and vomiting, or Prochlorperazine 10 mg IV every 6 hour prn nausea and vomiting
	Diet	Regular diet on POD0; oral hydration; gum chewing
	Foley catheter	Remove on POD1
	Activity	Ambulate 8 times a day; eat all meals sitting in a chair; stay out of bed 8 hours a day
	Transfusion	Restrictive; only for hemoglobin level > 7

Abbreviations: ER, endorectal; IV, intravenous; IVF, intravenous fluids; OR, operating room; PO, postoperative; POD, postoperative day; PRN, pro re nata (when necessary).

**Source:** Adapted from Miralpeix et al. (2015);^4^ Nelson et al. (2016);^7^ ^8^ Ljungqvist et al. (2017);^3^ and Carey and Molder (2018).^2^

The prophylaxis against thromboembolism includes well-fitting compression stockings and intermittent pneumatic compression. Extended prophylaxis (28 days) should be given to patients after laparotomy for abdominal or pelvic malignancies. The key postoperative protocol elements are early feeding (limiting the administration of intravenous fluids when the patient tolerates oral intake greater than 500 ml), early mobilization and opioid-sparing analgesia. A multimodal approach to postoperative nausea and vomiting with antiemetic agents should be used for patients undergoing gynecologic procedures. The patient should ambulate 8 times per day, have all meals sitting in a chair, and stay out of bed at least 8 hours per day.^4^ ^7^

## Final considerations

The implementation of the ERAS program represents a paradigm change in the perioperative management of the surgical patient, and is a multidisciplinary approach based on scientific evidence management.^3^ The program is clinically effective and has impacts on the outcomes of the patients, offering a safe, high-quality and cost-effective/cost-saving perioperative care. Therefore, the ERAS program should become the standard practice for all women undergoing elective gynecologic surgeries.^16^ Implementation challenges have been attributed to a variety of contextual factors, such as perceived lack of resources and resistance to change among providers. The number and combination of ERAS elements varied considerably across the studies. In Brazil, the challenge is to define strategies to adopt perioperative enhanced recovery programs in different scenarios. Compliance by the staff and the patients to the protocol elements of the ERAS is crucial to ensure a well-established and successful program.
